# Enrichment of Rapeseed Honey with Combined Polyphenol-Rich Dry Extracts from Mandarin Peels and Clove Buds

**DOI:** 10.3390/molecules31091487

**Published:** 2026-04-29

**Authors:** Małgorzata Dżugan, Monika Tomczyk, Alicja Strzałka, Michał Miłek

**Affiliations:** Department of Chemistry and Food Toxicology, Faculty of Technology and Life Sciences, University of Rzeszów, Ćwiklińskiej 1a, 35-601 Rzeszow, Poland; mdzugan@ur.edu.pl (M.D.); alicjastrzalka19@gmail.com (A.S.); mmilek@ur.edu.pl (M.M.)

**Keywords:** flavored honey, antioxidant, organoleptic properties, waste management, functional food

## Abstract

Enriching honey with plant additives allows for increasing its antioxidant potential in an additive-dependent manner and at the same time shaping new sensory properties, increasing consumer acceptability. Known spices and by-products from fruit processing can also be used to produce such additives. Combined dry extracts of clove buds and mandarin peels were used to enrich antioxidant properties and to flavor rapeseed honey. Four different extracts combining both raw products were produced by ultrasound-assisted extraction using 50% vol. ethanol and converted into powder by vacuum concentration followed by lyophilization. The obtained extracts were evaluated for antioxidant activity (FRAP and DPPH assays) as well as total polyphenols content. Phenolic HPLC-DAD profiles were compared and selected polyphenols (syringic acid, ellagic acid, hesperidin and eugenol) were quantified. The dry extracts were incorporated into rapeseed honey (0.25% *w*/*w*) during the creaming process. No significant changes in color and texture were visually noted; whereas, some changes (*p* < 0.05) in titrable acidity and electrical conductivity were observed. A significant increase (*p* < 0.05) in antioxidant activity (from 4 to 6-fold) and the beneficial enrichment with well-known bioactive compounds (mainly eugenol and hesperidin) was observed for all produced flavored honeys. Moreover, tested properties of the enriched honeys remained stable during 6 months of storage. The two honeys with the most improved antioxidant activity showed better sensory characteristics and consumer acceptability compared to pure rapeseed honey, but slight extract type-dependent differences were noted. It was shown that the proposed sustainable technological process using waste mandarin peels can lead to the development of a new product referred to as “plant-enriched honey” with increased health-promoting value belonging to the functional food segment.

## 1. Introduction

The rapid growth of functional food market encourages food technologists to search for innovative systems delivering bioactive compounds into human diet. Honey, traditionally known as a natural sweetener, is increasingly considered a promising carrier for bioactive substances in modern apitherapy and functional food development [1,2,3]. Despite its well-documented beneficial properties, light-colored honeys, especially rapeseed and acacia ones, have relatively low levels of polyphenols [4,5]. For this reason, enriching honey with additional ingredients may help create a more nutritionally valuable product. Rapeseed honey (*Brassica napus* L.) is often indicated as a suitable technological base for such products [2]. It is a pale honey, exhibiting low biological activity, including low antioxidant and antibacterial activity. Its relatively low price, exceptionally stable composition, fine crystallization and favorable rheological properties make it well suited for the production of stable creamed honey systems [6,7]. Moreover, its ability to temporarily become more fluid during mixing allows additives to be evenly dispersed in the honey without phase separation, which is an important requirement in industrial production [2].

Enriching nectar honey with plant additives increases its antioxidant potential, especially by raising the content of phenolic compounds and flavonoids. Such additives can strengthen the natural properties of honey, including its antibacterial, antiviral, and immunomodulatory effects [2,8,9]. Moreover, additives may provide easily digestible protein and important minerals such as potassium (K), calcium (Ca), magnesium (Mg), iron (Fe), and zinc (Zn), which are often lacking in the human diet [2,10]. And, most importantly, the natural sweetness of honey can mask the bitter or astringent taste of some medicinal plants like chokeberry, making such products more acceptable to consumers [11]. The use of different additives can also create new honey products with attractive color and aroma, which supports innovation in the beekeeping market [2,12,13].

A wide range of ingredients can be used to enrich honey, including wild medicinal plants (lungwort, primrose, elderflower, pine buds) [14,15], “superfruits” such as chokeberry, sea buckthorn, raspberry, blackberry, white mulberry, blackcurrant and apricot [8,11,16,17,18], as well as bee products like propolis, pollen, and bee bread [19,20]. Recently, the use of algae and halophyte plants, including spirulina, glasswort, and common purslane, has also gained attention [2,3]. The effect of enrichment is dependent on the type of ingredient. For example, additives such as spirulina or bee bread can increase the protein content in honey [2]. In some cases, plant additives (e.g., chokeberry or mulberry) can inhibit the activity of natural honey enzymes such as diastase [16,21]. Such products may also exhibit stronger antibacterial activity against pathogens such as *S. aureus* and *E. coli* [8,22]. Moreover, they may show antiviral potential, as demonstrated by the inactivation of model bacteriophages [8]. However, when selecting an additive, its potential toxicity should be taken into account, which is particularly important when using concentrated extracts, as well as its possible negative impact on the smell and color of the enriched product. In the case of pharmacologically active plants, the safety of enriched honey should be monitored. Also, the presence of heavy metals (e.g., lead or nickel) and microbiological contaminants which may be introduced with plant additives should be controlled [9,18].

Despite enhancing the antioxidant potential of honey, plant additives can affect the honey’s flavor which allows for the creation of new sensory characteristics in the final product to increase the interest of consumers seeking new flavors and products [13]. The selection of plant material for honey enrichment at the design stage of a new product allows us to predict both the degree of enhancement of antioxidant properties and changes in the product’s flavor and aroma. However, the development of a product’s sensory characteristics strongly depends on the form of the plant material used; thermal processing, such as drying, causes significant losses of volatile components in the raw material [23]. A good solution is to use essential plant oils as an additive [12] or to use appropriate extraction methods combined with concentrating the obtained extracts by removing the solvent [24]. Using dry plant extracts as honey additives minimally affects water content and visual characteristics of the honey (color and texture) [24] and allows for the use of inedible materials (clove buds) or even waste materials (mandarin peel). Utilization of the mandarin (*Citrus reticulata* Blanco) peel, treated as a by-product of the fruit processing industry, in food products supports the principles of sustainable development and waste reduction [25]. Moreover, due to its high content of aromatic compounds and bioactive substances, including flavonoids, phenolic acids, and essential oils, it may be considered a valuable raw material for creating the characteristic citrus aroma of honey-based products [26]. Thus, the combination of citrus fruit aroma with specific cloves aroma seems to be particularly promising and may therefore contribute to the development of a functional product with improved sensory properties.

The aim of the study was to produce dry extracts based on cloves (*Syzygium aromaticum* (L.) Merr. and Perry) and mandarin (*Citrus reticulata* Blanco) peels using various extraction systems, and to compare their antioxidant properties and polyphenolic profile. For the first time, the most abundant extracts were used to enrich rapeseed (*Brassica napus* L.) honey. The degree of enhancement of the antioxidant properties and polyphenol profile of the enriched honeys, their storage stability, and consumer acceptability were determined.

## 2. Results and Discussion

Both mandarin peels and clove buds were extracted with 50% ethanol separately (crude M and C extracts) and in a 1:1 *w*/*w* mixture (M+C variant). Furthermore, the effect of a subsequent extraction was tested, where previously prepared M peel extract was used for leaching the clove buds (b+M variant), and the mandarin peels were extracted with a ready-made C extract (p+C variant). The liquid extracts were converted to powders by a 2-step concentration process: (1) vacuum evaporation of the alcohol and (2) freeze-drying.

### 2.1. Antioxidant Properties of Obtained Crude Extracts

**Figure 1:**
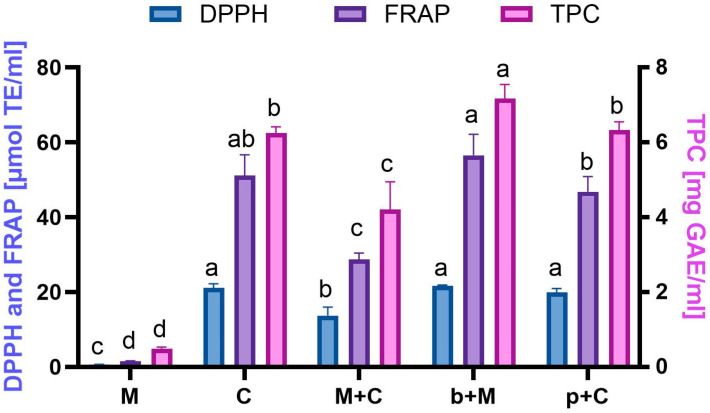
Antioxidant activity measured by FRAP and DPPH assays and TPC in tested crude extracts. ^a,b,c,d^—different superscript letters within the same assay indicate statistically significant differences between the analyzed samples (*p* < 0.05). M—mandarin peel extracted with 50% ethanol; C—clove buds extracted with 50% ethanol; M+C—mandarin peel and clove buds (1:1) extracted with 50% ethanol; b+M—clove buds extracted with M extract; p+C—mandarin peel extracted with C extract.

The results of the study (Figure 1) showed that the crude clove extract (C) significantly exceeded (*p* < 0.05) the antioxidant activity of the mandarin extract (M), by approximately 30-fold in both DPPH and FRAP methods. At the same time the total phenolic content (TPC) in the C sample was about 13-fold higher than in M extract.

Mixing the raw materials before extraction had a beneficial effect on the antioxidant activity of the M+C extract, resulting in intermediate values in all applied assays. In contrast, performing subsequent extraction (b+M) significantly increased the DPPH antiradical activity to 21.68 µmol Trolox/mL, making it the sample with the highest antioxidant activity among all tested samples. In the opposite approach, when the previously prepared clove extract was used to extract mandarin peels (p+C), the resulting extract showed lower antioxidant activity than b+M, but still higher than the extract obtained from the direct extraction of the raw material mixture (M+C). The FRAP results confirmed these observations, with the reducing power of the extracts decreasing in the following order: b+M > C > p+C > M+C >> M. Similar trends were observed for the total phenolic content (TPC), where the clove extract contained significantly higher levels of phenolic compounds (*p* < 0.05) than the mandarin extract. As in the previous assays, extraction of the raw material mixture (M+C) resulted in a lower polyphenol content compared with the subsequent extraction, by approximately 1.7-fold and 1.5-fold for the b+M and p+C extracts, respectively.

The higher antioxidant activity and phenolic content observed in clove extracts compared to mandarin extracts can be attributed to the presence of eugenol—specific phenolic compounds of cloves. This dominant phenolic compound exhibits strong antioxidant and anti-inflammatory properties. In contrast, phenolic compounds present in mandarins, such as hesperidin and naringenin, also demonstrate antioxidant activity, although much weaker than that of eugenol [27,28]. The increased antioxidant activity observed in the b+M preparation may indicate the occurrence of synergistic interactions between phenolic compounds originating from mandarins and cloves. It is likely that phenolic compounds from mandarin peels complement the spectrum of phenolic compounds present in cloves, leading to more efficient extraction of antioxidant constituents. On the other hand, the lower antioxidant activity observed for the p+C extract may result from limited extraction of peel polyphenols when clove extract, already rich in phenolic compounds, was used as the extraction medium. It is well known that the use of a solvent phase already saturated with extractable compounds can reduce the efficiency of further extraction processes [21].

### 2.2. HPLC Polyphenol Profiles of Crude Extracts

**Figure 2:**
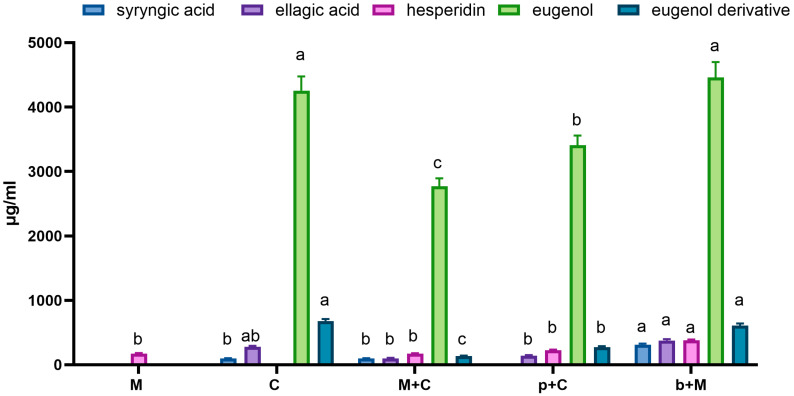
Results of quantitative analysis of selected polyphenols in crude extracts by HPLC-DAD. ^a,b,c^—different superscript letters within the same assay indicate statistically significant differences between the analyzed samples (*p* < 0.05). M—mandarin peel extracted with 50% ethanol; C—clove buds extracted with 50% ethanol; M+C—mandarin peel and clove buds (1:1) extracted with 50% ethanol; b+M—clove buds extracted with M extract; p+C—mandarin peel extracted with C extract.

For extracts converted to powder by two-step concentration process the polyphenolic profile was established (Table 1; Figure S1) and the main polyphenols were quantified (Figure 2) by HPLC analysis.

Comparison of the chromatographic profiles of extracts from individual raw materials (M and C samples) allows us to identify their specific phenolic compounds (Table 1). In the case of the mandarin extract, the primary identified compounds were phenolic acids (including chlorogenic acid isomers and other unrecognized caffeic acid derivatives) and flavonoids (characteristic hesperidin and numerous derivatives of luteolin, quercetin, apigenin, and kaempferol). In the extract of clove buds, the dominant metabolites were eugenol (and its derivative), as well as phenolic acids: ellagic, syringic, gallic and numerous precisely unidentified derivatives, characterized by UV/Vis spectra similar to those of gallic acid. These observations are similar to the results for analogous extracts reported in the literature. In mandarin peel extracts, hesperidin was reported as the dominant compound, along with other flavonoids, including narirutin, naringin, rutin, taxifolin, and nobiletin [29,30]. In the case of cloves, studies have shown that eugenol constitutes up to over 50% of all identified compounds in clove extracts and essential oils [31,32]. Eugenol acetate, which may be a derivative identified in the tested extracts, is also usually determined in large amounts [31]. Moreover, the literature data confirm the presence of numerous phenolic acids, including gallic, ellagic, and syringic acids, and flavonoids, e.g., kaempferol, quercetin, myricetin and their derivatives [33,34]. The use of extraction from two raw materials simultaneously resulted in the recovery of most of the main metabolites; however, the subsequent extraction method using a crude extract of one of the raw materials causes greater changes in the qualitative composition of the final extract. By extracting cloves with mandarin peel extract, the qualitative composition is rich, and the extract obtained (b+M) also contains most of the components identified in cloves and mandarin peel. In the case of the reverse extraction (p+C), a specific inhibition of the recovery of certain compounds (phenolic acids, including syringic acid and other gallic acid derivatives as well as some flavonoids) from mandarin peel was observed.

The extraction efficiency of selected compounds in various extraction systems was also quantitatively assessed (Figure 2), including hesperidin—a dominant flavonoid from mandarin—and syringic acid, ellagic acid, and eugenol (with a derivative) as cloves representative. In the M extract, hesperidin was determined at 172.7 µg/mL and an almost identical concentration was determined for the M+C extract (171.4 µg/mL). In the case of subsequent extracts, more of this compound was determined; however, there is a possibility of overlapping of other components derived from cloves and eluted at a similar retention time. Eugenol, the main phytochemical component of cloves, was present in the crude extract at 4.25 mg/mL. Similar levels were found in the p+M extract (*p* > 0.05), while those in the M+C and b+M extracts were lower, by 35% and 20% respectively. A similar trend was observed for the eugenol derivative being assayed. It can be concluded that certain mechanisms limit the recovery of these compounds when using mandarin extract instead of pure solvent. A similar negative effect on the extraction efficiency of berberine when using subsequent extraction of barberry root with propolis extract instead of pure alcohol was observed earlier [35]. In the case of subsequent extraction, final effect is probably the result of two simultaneous processes: extraction of components from the raw material and surface bonding of the extraction solvent components by the raw material.

**Figure 3:**
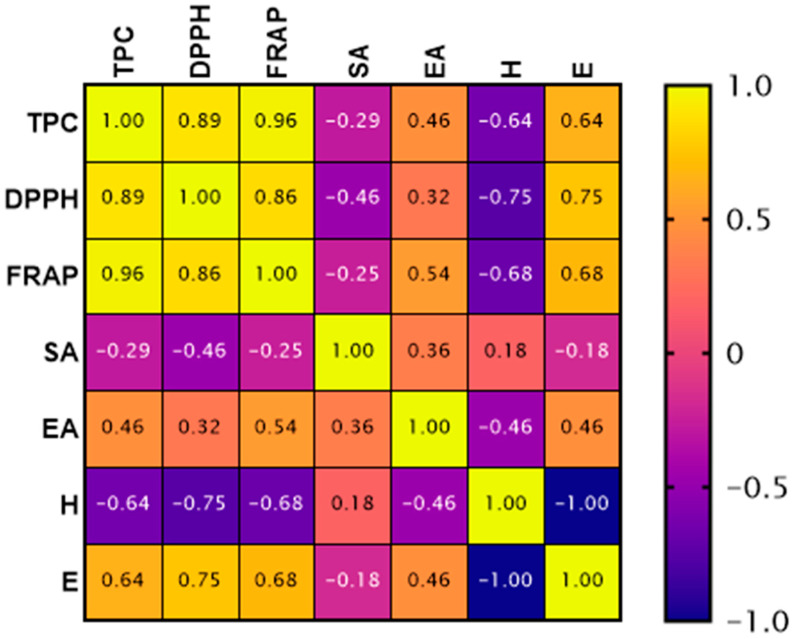
Spearman’s rank correlation matrix between tested parameters for crude extracts.

The correlation analysis (Figure 3) revealed very strong positive relationships between total phenolic content (TPC) and antioxidant activity measured by FRAP (r = 0.96) and DPPH (r = 0.89) method, confirming that phenolic compounds are the primary contributors to the antioxidant potential of the analyzed extracts. Among the individual phenolic compounds, eugenol showed the strongest positive correlations with antioxidant parameters (DPPH r = 0.75; FRAP r = 0.68; TPC r = 0.64), suggesting its dominant role in the antioxidant activity of clove-containing extracts. This strong effect may be attributed to the phenylpropanoid structure of eugenol, which facilitates efficient radical scavenging and electron donation [36]. Ellagic acid showed moderate positive correlations with FRAP (r = 0.54) and TPC (r = 0.46), while syringic acid exhibited weak or negative correlations with the analyzed antioxidant parameters. In contrast, hesperidin showed strong negative correlations with antioxidant activity (DPPH r = −0.75; FRAP r = −0.68; TPC r = −0.64), indicating a lower contribution of mandarin-derived flavonoids to the overall antioxidant potential. Similar relationships between phenolic composition and antioxidant activity have been reported previously for plant extracts rich in phenylpropanoids and flavonoids [27,28].

### 2.3. Physicochemical Properties of Enriched Honeys

Based on the evaluated extracts activity, four variants of enriched honey were prepared: H_M+C,_ H_b+M,_ H_p+C_, and H_M&C,_ each time with the use of 0.25% of dry extracts: M+C, b+M, p+C and combination of M and C (M&C) in the ratio 1:1 (*w*/*w*). After 1 month of storage, enriched honeys were tested regarding selected physicochemical parameters and compared to pure honey (H) (Table 2). Results showed that the addition of all extracts slightly reduced the pH of the honeys compared to the control honey (*p* > 0.05). At the same time, the enriched honeys were characterized by lower acidity than the control rapeseed honey, however the changes depended on the type of extract used. In the case of both combined b+M and p+C extracts, significant decreases in acidity were observed (*p* < 0.05). The addition of extracts to all tested honeys resulted in increased electrical conductivity compared to the control honey and significant changes in the case of H_b+M_ and H_M&C_ were observed (*p* < 0.05). Phenolic compounds introduced in extracts have weak acidic properties and dissociate in solutions with the release of H^+^ ions [37]. Other organic acids may also be present in the extracts. It may explain the decrease in the pH of the product. The increase in electrical conductivity is likely associated with the higher content of mineral components and phenolic substances introduced with the extracts, which increase the ionic strength of the honey matrix, as reported previously for plant-enriched honeys [2,25].

### 2.4. Antioxidant Properties of Enriched Honeys

The antioxidant properties of the enriched honeys were tested after one month of storage using standard colorimetric assays compared to control honey stored at the same conditions (Table 3). To assess the effect of the additive on the honeys’ antioxidant properties, the enrichment degree was determined relatively to the control honey, which was taken as a reference value (100%). All enriched honeys showed significantly higher antioxidant potential (*p* < 0.05) compared to rapeseed honey and the enrichment grade depended on the initial activity of the added extract. Antioxidant properties analysis of the enriched honeys using the FRAP method revealed a significant enrichment compared to the pure honey, ranging from 662 to 1032% and the highest increase was observed for the honey with the M&C extract. Antiradical activity tested using the DPPH method revealed a significant increase compared to the base honey, ranging from 349 to 509% (Table 3). The highest value was also recorded in honey enriched with M&C extract. All enriched honeys were characterized by a significantly higher ability to neutralize DPPH radicals (*p* < 0.05) and the ability to neutralize free radicals depended on the initial activity of the added additive. Enrichment of honeys with mandarin and clove extracts led also to a significant increase in phenolic compound concentrations compared to the control honey, ranging from 330 to 395%. The largest increase was observed in samples with b+M extract. Statistical analysis confirmed a significant increase in the total phenolic compound content in the enriched honeys and the observed increase significantly depended on the activity of the added extract (*p* < 0.05).

Enriching honey with natural additives as a promising modification for increasing its antioxidant properties has been previously reported. For honey enriched with freeze-dried blackberry and raspberry fruits (4%), the antioxidant potential increased 4-fold [8]. Similar effects were observed in honey enriched with mulberry leaves where the antioxidant activity increased almost 4-fold compared to raw rapeseed honey [16]. Moreover, the observed effect was dose-dependent, the higher share of mulberry leaves the higher enrichment of antioxidant properties was observed, reaching even a 50-fold increase in antioxidant activity (DPPH) for the 4% addition [16]. The addition of 4% dried *Primula veris* flowers has been reported to increase the DPPH radical scavenging activity of honey by more than 50-fold, indicating the high enrichment potential of this plant resulting from its rich phenolic composition [14]. Moreover, cited studies assessed the total content of polyphenolic compounds in enriched honeys, and a high correlation (r = 0.980–0.990) between polyphenols content and the antioxidant activity of the honeys was found. The authors suggested that phenolic compounds play a significant role in shaping the antioxidant properties of honeys.

### 2.5. HPTLC Polyphenol Profiling of Enrichment Honeys

**Figure 4:**
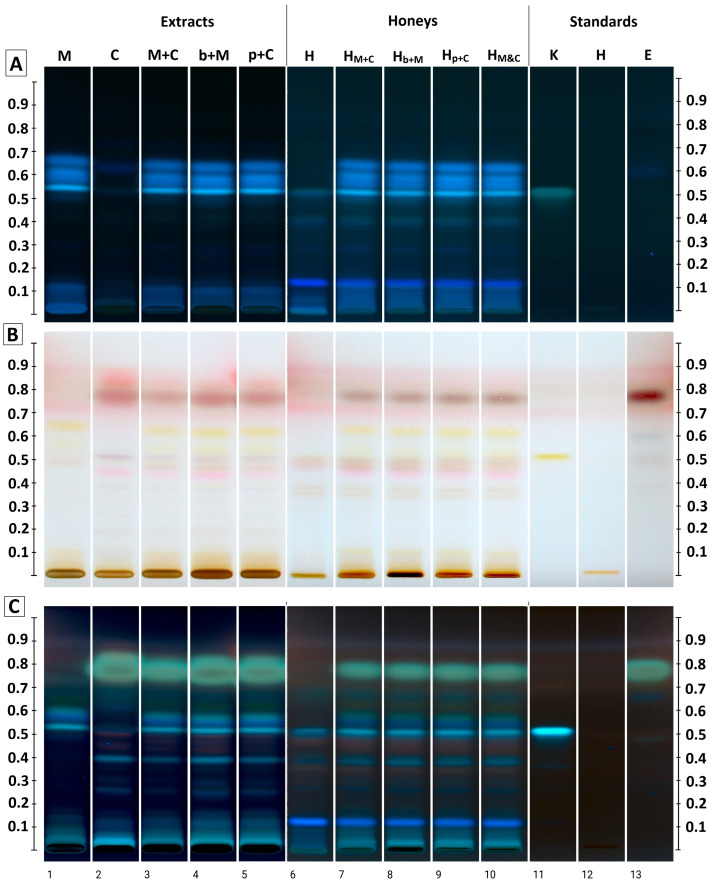
HPTLC results for tested extracts (tracks 1–5) and 1-month stored honey extracts after SPE (tracks 6–10), as well as standards (tracks 11–13). (**A**)—plate before derivatization, image in UV 366 nm; (**B**)—plate after p-anisaldehyde reagent derivatization, image in white light; (**C**)—plate after p-anisaldehyde reagent derivatization, image in UV 366 nm; K—kaempferol; H—hesperidin; E—eugenol.

To assess the enrichment of rapeseed honey in compounds derived from the introduced extracts HPTLC method was used, which allows for easy comparison of the phytochemical profiles of multiple samples simultaneously. An image of the HPTLC plate before and after derivatization with p-anisaldehyde reagent is shown in Figure 4. In the comparison of crude extracts alone, the blue bands at Rf 0.11, 0.53, 0.58, 0.60 and 0.66 dominate in the UV 366 nm and were identified corresponding to compounds derived from the mandarin peel. However, their different coloration under the influence of the derivatizing agent indicates their different chemical nature. In the clove extract, a strongly overloaded band due to eugenol (Rf = 0.78) is visible. This is accompanied by weaker bands in the Rf range between 0.25 and 0.4, which were also detected in the combined extract samples. In the case of the b+M and p+C extracts, the track images are almost identical—difficult to distinguish using this method. The band pattern was identical, with only the intensity of selected bands (Rf = 0.4 and the eugenol band) being slightly lower in the p+C extract. The M+C extract also showed the presence of all bands, both from orange mandarin peel and clove buds. However, the intensity of the bands corresponding to clove-derived compounds (including eugenol) after derivatization was lower, confirming the lower extraction efficiency of bioactive compounds in this variant, as observed using other methods.

Bands originating from the introduced extracts were also intensely visible in the tracks corresponding to the analyzed honeys (SPE extracts). Among the compounds originating from rapeseed honey, kaempferol (Rf = 0.52, blue-green color in UV 366, yellow in white light after derivatization) and an intense navy blue band at Rf = 0.12, characteristic of this honey variety, are worth mentioning [12]. All the bands observed in the extract chromatograms were visible in the enriched extracts, and their intensity was also so similar in all cases that this method cannot indicate which profile is richer. Similar enrichment of honey, mainly in eugenol, was previously observed in the case of the addition of clove essential oil [12]. Hesperidin from mandarin peel remains at the starting point in applied chromatographic system, but it develops a characteristic orange color (under white light) and black color (under UV 366). Its presence is visible in both extracts and enriched honeys.

The HPTLC method has previously been successfully used to assess the degree of enrichment of honey with phytochemicals as a result of introducing plant additives. Enrichment with anthocyanins and flavonoids was demonstrated using this method as a result of adding chokeberry, raspberry, and blackberry fruits, as well as *Rubus* spp. leaves [8,11]. The usefulness of the method for rapid detection of honey enrichment with plant polyphenols was confirmed once again in this study; however, it is a good tool for qualitative screening; quantitative assessment analysis by another method (e.g., HPLC) is necessary.

### 2.6. HPLC Analysis of Polyphenols in Enriched Honeys

Control rapeseed honey (H) and samples enriched with extracts were also analyzed using HPLC-DAD. The samples were prepared using the commonly used SPE method, which allows for the concentration of analytes while simultaneously removing ballast substances, primarily sugars in the case of honey. A comparison of the chromatograms for the analyzed honeys (on the same scale) is presented in Figure S2.

The profile obtained for the control honey was typical of this honey variety from Central and Eastern Europe. The identified compounds included primarily phenolic acids and their derivatives—p-coumaric acid, ferulic acid, benzoic acid, methyl syringate—as well as flavonoids kaempferol, pinobanksin, and pinocembrin (Table 4). The above-mentioned identified compounds were also previously detected in Polish rapeseed honey as dominant polyphenols [38,39]. The compound that gave the most intense signal was methyl syringate, similarly to what was described by Kuś et al. [40]. Honey-derived compounds were also detected in honey samples enriched with extracts, with the exception of pinocembrin. Among the phytochemicals extracted from orange mandarin peel and cloves, several were identified, with a clear predominance of eugenol. Compared to previous analysis of the extracts used the polyphenols profiles of enriched honey revealed the presence of a smaller number of compounds was observed (Table 1). No signals were detected from substances such as gallic acid, chlorogenic acid isomers, and numerous derivatives of phenolic acids and flavonoids. This may be due to losses during extract concentration and freeze-drying or during honey purification using SPE. Generally, the sample preparation methods used are mild with respect to phenols; however, in some cases they may lead to losses of specific compounds due to the effects of temperature or interactions with the sorbent during SPE [41,42]. Probably, these compounds were present in the final product at concentrations below the detection threshold of the chromatographic method used.

**Figure 5:**
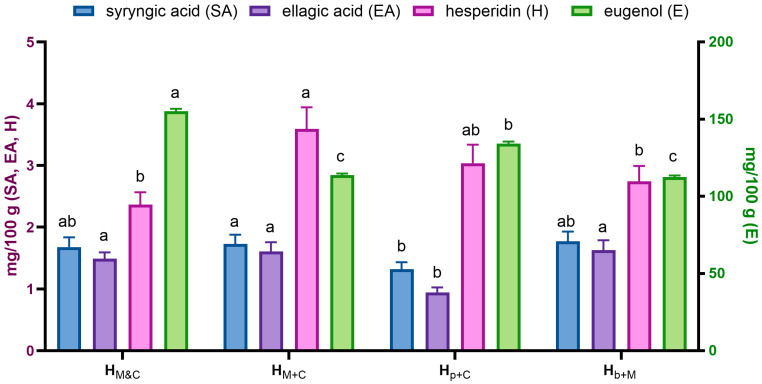
Quantitative analysis of selected phytochemicals in honeys enriched with mandarin and clove extracts by HPLC-DAD performed after 1 month of storage. The right Y-axis refers to eugenol, the left to the remaining compounds. ^a,b,c^—different superscript letters within the same compound indicate statistically significant differences between the analyzed samples (*p* < 0.05).

As with the crude extracts, the same compounds were quantified (Figure 5), with the exception of the eugenol derivative, which was not detected in any sample.

In all cases, honey was strongly enriched in eugenol (112–155 mg/100 g). The remaining quantified compounds were present at lower concentrations (up to 3 mg/100 g). Interestingly, when C and M dry extracts were added as mixture (sample H_M&C_), the eugenol content was significantly higher than when the M+C extract was added to honey. For hesperidin, the trend was reversed. The profile of analyzed phenolic compounds in honeys enriched with dry extracts does not accurately reflect their content in raw liquid extracts. Higher eugenol contents were observed for the b+M sample than for the p+C extracts whereas for enriched honey samples adverse tendency was observed. This may indicate differentiated losses of this volatile compound during conversion of liquid extract to dry form, which may result from interactions between the components originated from mandarin and clove, but this requires further research.

The enrichment of the honey with phytochemicals derived from a plant additive (in the form of freeze-dried fruit or essential oil) has been previously described [8,12,16,43]. The addition of various freeze-dried fruit, including orange, to rapeseed honey resulted in the appearance of new polyphenols in the product, such as quercetin glycosides and protocatechuic acid [44]. Orange addition introduced, among others, neochlorogenic and protocatechuic acids, and also increased the concentration of compounds already present in the initial honey, such as caffeic and p-coumaric acid. Functional products created by blending honey with selected dried fruits were also assessed for their polyphenol content by Geană et al. [45]. The authors noted significant enrichment of honey in chlorogenic acid, gallic acid, rutin, epicatechin, and even naringin and resveratrol.

### 2.7. Storage Stability of Enriched Honeys

**Figure 6:**
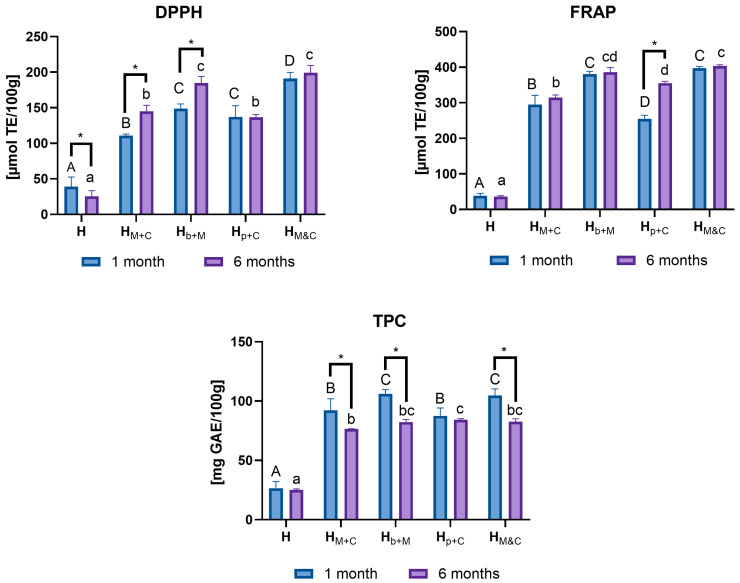
Changes in antioxidant activity of enriched honey vs. pure honey (H) after prolonged storage. *—statistically significant differences (*p* < 0.05) between time of storage (1 month vs. 6 months); ^A,B,C,D^—different superscript letters within honey samples indicate statistically significant differences (*p* < 0.05) after 1 month of storage; ^a,b,c,d^—different superscript letters within honey samples indicate statistically significant differences (*p* < 0.05) after 6 month of storage. H—control honey; H_M+C_—honey enriched with M+C dry extract; H_b+M_—honey enriched with b+M dry extract; H_p+C_—honey enriched with p+M dry extract; H_M&C_—honey enriched with combination M and C extracts in the ratio 1:1.

Honeys stored for 6 months at room temperature were analyzed for antioxidant properties (FRAP and DPPH) and phenolic content in comparison to the values obtained for honeys evaluated after 1 month of storage (Figure 6). It was observed that honey with added extracts retained or even increased its antioxidant activity measured by FRAP test. During storage, the content of phenolic compounds in the honeys studied decreased slightly. Stability of antioxidant parameters (FRAP and DPPH) and total phenolic in plant-enriched honeys during storage, which have been reported previously, depended on the type of additive, its form, and the duration of storage. High stability of phenolic compounds and antioxidant activity for honeys enriched with spirulina [2] or essential oils (e.g., cinnamon or clove) [12] were observed for up to six months. In honey enriched with chokeberry extracts, antioxidant parameters even initially increased during the first weeks of storage [11]. Slight decreases in TPC and antioxidant activity have also been observed in honeys enriched with raspberry or blackberry leaves and fruits after three months of storage [8]. These changes are mainly related to natural degradation processes, interactions between honey components and plant additives, and the gradual stabilization of the physicochemical system, indicating that the changes depend on the type of additive introduced to the honey. Studies of changes in individual polyphenol content during honey storage are rarely conducted due to numerous factors potentially influencing the polyphenol profile, including pH, temperature, enzymatic activity, and possible interactions between honey components. Šarić et al. studied the content of eight flavonoids in two honey varieties during 12 months of storage. They demonstrated differential behavior of individual flavonoids; in general, the content of flavonoids in honey samples increased between the 1st and 6th months of storage and then began to decrease until the 9th month, when they remained relatively constant throughout the 12th month of storage [46].

### 2.8. Organoleptic Assessment Results

Two raw materials are used to enhance the honey’s health-promoting properties: clove buds, known for their antibacterial properties [32], and waste mandarin peels, a rich source of flavonoids [47], were intended also to create attractive sensory characteristics to the designed products. Both used raw materials were characterized by a high content of specific volatile compounds responsible for the spicy aroma of cloves [48] and the fruity aroma of mandarin peels [26].

**Figure 7:**
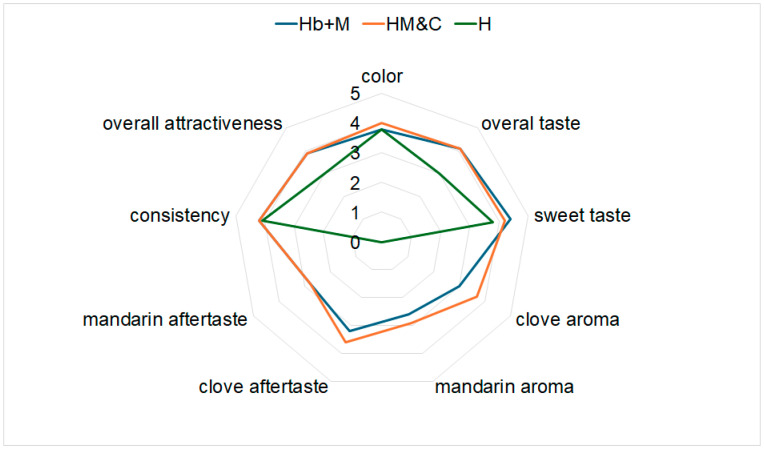
Results of organoleptic evaluation for selected honeys after 6 months of storage using five-point scale.

After six months of storage, a sensory evaluation was conducted on honeys enriched with mandarin and clove extracts exhibiting the best antioxidant properties: H_b+M_ and H_M&C,_ compared to pure rapeseed honey. A group of 10 trained people participated in the tasting, assessing five main criteria: color, taste, aroma, consistency, and overall attractiveness. Additionally, due to the addition of citrus and herbs, the aroma and flavor of mandarin and clove, as well as overall sweetness, were assessed in detail. The evaluation results are presented in Figure 7. Overall assessment was comparable for both enriched honey samples; however, it was much more beneficial for the enriched than for the pure honey, indicating that the combination of mandarin peel and cloves extracts positively influenced the sensory acceptance of the product without affecting texture. Although, both enriched honey samples were assessed similarly, the panelists indicated some differences regarding a more pronounced clove aroma and flavor for H_M&C_ sample compared to H_b+M_. Interestingly, this difference in organoleptic evaluation is justified by the results of the HPLC analysis, which revealed a higher eugenol content (by 39%) in the H_M&C_ sample than in the H_b+M_ sample (Figure 5). These results suggest that the use of obtained dry extracts can enhance the sensory characteristics of rapeseed honey and contribute to the development of novel honey-based products with distinctive flavor and aroma profiles.

The introduction of plant additives significantly modifies the sensory profile of honey, affecting its color, flavor, aroma, and texture [9,12]. Additives rich in natural pigments typically darken the product (decrease in the lightness parameter L*) and impart new shades [21]. For example, chokeberry dyes honey purple-red, while spirulina can impart an intense green or blue color [2,11]. Honey also serves as a good carrier matrix for aromatic additives, as its natural sweetness masks the bitter or astringent taste of many plant ingredients [3,11]. Spices such as cinnamon, ginger, and cardamom impart intense spicy notes to honey and can reduce the perception of sweetness [13]. However, higher concentrations, like the addition of propolis above 0.5%, can lead to an overly sharp, bitter taste, and reduced consumer acceptance [20]. In technological practice, optimal additives allow for the creation of innovative products with attractive sensory characteristics while maintaining high consumer acceptance [2,12,21].

## 3. Materials and Methods

### 3.1. Material and Chemicals

The raw materials used to obtain the extracts were mandarin fruit peels (*Citrus reticulata* Blanco) self-obtained from fruits purchased in a local store (country of origin: Spain) and clove buds (*Syzygium aromaticum* (L.) Merr. and Perry) packaged by Prymat (Jastrzębie Zdrój, Poland). Rapeseed honey produced by honeybees (*Apis mellifera carnica*) came from a local apiary (N 50°31′, E 21°28′) and was harvested in the 2024 beekeeping season. All chemicals used were at least of analytical grade. Solvents (ethanol, acetonitrile, methanol, chloroform, ethyl acetate) were purchased from Honeywell (Charlotte, NC, USA), chemicals for colorimetric analyses (Folin–Ciocalteu reagent, Na_2_CO_3_, FeCl_3_, TPTZ, DPPH, Trolox, Natural Product reagent, PEG 400) as well as standards (gallic acid, ellagic acid, syringic acid, p-coumaric acid, chlorogenic acid, neochlorogenic acid, cryptochlorogenic acid, ferulic acid, luteolin, rutin, kaempferol, hesperidin, pinobanksin, pinicembrin, methyl syringate, eugenol) came from Sigma-Aldrich (St. Louis, MO, USA).

#### 3.1.1. Plant Dry Extracts Preparation

**Figure 8:**
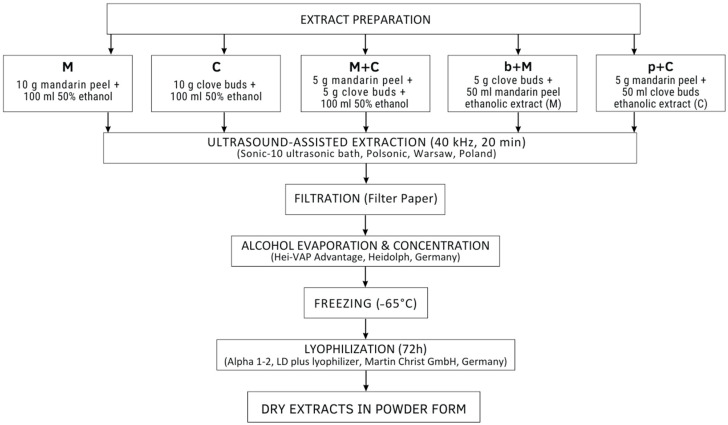
Scheme of preparation of mandarin peel and clove dry extracts.

Mandarin peels were grated, and clove buds were crushed in a porcelain mortar. Extracts from mandarin peels (M), clove buds (C), and their combinations were prepared according to the procedure presented in Figure 8. The prepared samples were subjected to ultrasound-assisted extraction using an ultrasonic bath (Sonic-10, Polsonic, Warsaw, Poland) at a frequency of 40 kHz for 20 min. Subsequently, the extracts were filtered through fluted filter paper. The obtained filtrates were concentrated by evaporating ethanol under reduced pressure using a vacuum evaporator (Hei-VAP Advantage, Heidolph, Germany). The remaining residue was frozen at −65 °C and then lyophilized for 72 h using an Alpha 1–2 LD plus freeze dryer (Martin Christ GmbH, Osterode, Germany) to obtain dry extracts in powder form.

#### 3.1.2. Enriched Honey Preparation

To enrich rapeseed honey, dry extracts in powder form (M+C, b+M, p+C as well as mixture of M and C extracts (in the mass ratio 1:1) were used; the applied amount of additive (0.25% *w*/*w*) were chosen in preliminary studies based on organoleptic evaluation of small-batch samples prepared with increased additive share (0.1, 0.25 and 0.5%). For the selected concentration, the aroma of the additives was noticeable but was not assessed as too strong and artificial. The procedure of honey enrichment was carried out as previously described by [2]. Briefly, the honey in a glass jar was liquefied by heating in a laboratory incubator for 24 h at 40 °C. Then, 99.75 g of honey was weighed into 200 mL glass jars using an analytical balance, and 0.25 g of each variant of dry extracts were added, obtaining four products: H_M+C_, H_b+M_, H_p+C_, and H_M&C_. After adding the honey additive, each sample was thoroughly mixed with a hand mixer at low speed for 4 min to evenly distribute the additives throughout the enriched honey. The prepared samples were then placed in a refrigerator at 4 °C for 24 h. The products were stored refrigerated for 14 days and then at room temperature, away from sunlight and moisture, for six months. Analysis of honey parameters was performed after one and six months of storage. For analysis of physicochemical parameters and antioxidant capacity 20% (*w*/*v*) aqueous solutions of honey samples were prepared.

### 3.2. Methods

#### 3.2.1. Total Phenolic Content

The determination of the total content of polyphenolic compounds in the obtained extracts and aqueous solutions (20%) of control and enriched honeys was carried out using the method described in Miłek et al. [11]. The polyphenolic compound content was expressed as gallic acid equivalents [mg GAE/100 g honey], calculated from a gallic acid standard curve (y = 0.341x, R^2^ = 0.9974), prepared over a concentration range of 0–125 μg/mL.

#### 3.2.2. Antioxidant Properties

The analysis of the antioxidant capacity of extracts and aqueous solutions of control and enriched honeys was performed using the FRAP and DPPH methods, according to the procedure described by Miłek et al. [11].The FRAP results were expressed as trolox equivalents (μmol TE/100 g honey), were calculated based on a Trolox standard curve (y = 0.152x, R^2^ = 0.9989), prepared over a concentration range of 5–60 nmol/mL.

The DPPH results were calculated as percentage of free radical scavenging and then converted into Trolox equivalents [μmol of Trolox per 100 g of honey], using the standard curve equation described by the equation: y = 15.553x (R^2^ = 0.9970), where x is the determined value of % inhibition of the previously obtained results.

#### 3.2.3. HPLC Polyphenols Analysis

To prepare samples for chromatographic analyses, honeys were subjected to solid-phase extraction, removing sugars and concentrating polyphenols [8], using Sep-Pak Plus Short C18 columns (Waters). The extraction procedure was carried out by passing through the columns sequentially 10 mL of methanol, 10 mL of acidified water (pH 2 adjusted with 1M HCl), 100 mL of the test sample (20% solution of enriched honey in acidified water), 10 mL of acidified water (pH 2), and 5 mL of methanol. The methanolic fraction was subjected to chromatographic analysis.

Chromatographic analysis was performed using a Gilson chromatographic system (HPLC chromatographic system, Gilson, Inc., Middleton, WI, USA), which included a binary pump (Gilson 322), a column thermostat (Knauer, Berlin, Germany), an autosampler with a fraction collector (Liquid Handler GX-271), and a diode array detector (DAD, Gilson 172, Gilson, Middleton, WI, USA). Samples were separated using a Poroshell 120, EC C-18 analytical column, 4.6 × 150 mm (Agilent Technologies, Santa Clara, CA, USA), with the column temperature maintained at 40 °C. The chromatographic process was performed in gradient mode using 0.1% (*v*/*v*) formic acid in water as phase A and acetonitrile as phase B, at a mobile phase flow rate of 1 mL/min. A gradient program was used: 10% B for 1.5 min, increasing from 10% to 100% B over 1.5–20 min, holding at 100% B for 20–25 min, then returning to 10% B to equilibrate the column. Ten microliters of 10-fold diluted samples was injected, and chromatograms were recorded at λ = 280 nm. Phenolic compounds were identified based on UV-VIS spectra and retention time comparison with analytical standard values, supported by literature data. In order to determine the concentration of selected identified compounds: syringic acid, hesperidin, ellagic acid and eugenol (along with its derivative) in the tested extracts, standard curves were determined for each compound using analytical standards (Sigma Aldrich, Saint Louis, MO, USA). Calibration was performed using chromatograms collected at 280 nm. For the quantification of eugenol, the extracts were diluted 5-fold. Method validation data is provided in Table S1.

#### 3.2.4. HPTLC Analysis

Chromatographic analysis was performed using a HPTLC system, which included an automatic applicator (Linomat 5) for precise banding, a developing chamber (ADC2) for controlled plate development, a visualizer, and a derivatizer for band visualization (Camag, Muttenz, Switzerland). SilicaGel 60 F254 HPTLC plates (Merck, Darmstadt, Germany) were used to determine polyphenolic compounds. Three microliters of extract were applied as bands 8 mm wide from the bottom edge of the plate. Extracts were applied at a rate of 100 nL/s using a semiautomatic HPTLC applicator. Additionally, the following standards (250 μg/mL in methanol) were applied: kaempferol, hesperidin and eugenol (Sigma Aldrich, Saint Louis, MO, USA). The separation of polyphenolic compounds was performed in a chromatographic chamber saturated with the mobile phase (chloroform, ethyl acetate, formic acid 5:4:1) for 20 min. The obtained results were imaged using an HPTLC imager at wavelengths of 254 and 366 nm. Finally, the plate was derivatized with p-anisaldehyde using an automated TLC derivatizer. After this process, the plates were imaged under visible and UV 366 nm light. The obtained chromatographic images were analyzed using HPTLC software (Vision CATS 3.2, CAMAG, Muttenz, Switzerland) [11].

#### 3.2.5. Physicochemical Evaluation of Enriched Honeys

Electrical conductivity was determined using a 20% (*w*/*w*) honey solution by immersing the glass electrode of a CP-401 conductivity meter (Elmetron, Zabrze, Poland). The measurement was recorded in mS/cm after the reading had stabilized. Each determination was performed in triplicate.

Active acidity (pH) was determined using an advanced pH meter (Seven Compact, Mettler-Toledo, Columbus, OH, USA) equipped with a combined glass electrode. Free acidity was determined by titration method [11]. Each determination was carried out in duplicate and the final results were expressed as milliequivalents per kilogram of honey (mEq/kg).

#### 3.2.6. Organoleptic Assessment

A sensory evaluation was performed for selected honeys enriched with dry extracts characterized by the highest antioxidant activity and pure honey. The analysis was conducted after six months of storage. The evaluation was carried out by a panel of 10 trained assessors aged 24–25 years. Each sample was coded to ensure an unbiased assessment. The panel evaluated the samples according to the following attributes: color, overall flavor, sweet taste, clove aroma, mandarin aroma, clove aftertaste, mandarin aftertaste, consistency, and overall desirability. The assessment was conducted using a five-point scale, where 1 indicated the lowest score (unacceptable product) and 5 the highest score (very well accepted product). Detailed scoring criteria established for each attribute were as follows: for color from 1 (very unattractive, unusual) to 5 (very attractive, characteristic); overall flavor from 1 (unacceptable) to 5 (very good, well-balanced); sweetness from 1 (very weak or excessive) to 5 (optimal and well-balanced); aroma (cloves and mandarin) from 1 (none or unpleasant) to 5 (intense and characteristic), and aftertaste from 1 (none or unpleasant) to 5 (long-lasting and pleasant); consistency from 1 (very weak, unusual) to 5 (typical of honey). Overall desirability was rated from 1 (unacceptable) to 5 (highly desirable).

### 3.3. Statistical Analysis

All measurements were performed in triplicate unless otherwise stated, and the results are presented as mean ± standard deviation (SD). Statistical differences among samples, including the type of extract used as a honey additive and the storage time, were assessed using multivariate analysis of variance (ANOVA) followed by Tukey’s post hoc test (*p* < 0.05). Spearman’s rank correlation analysis was applied to determine relationships between the analyzed parameters. Statistical analyses and data visualization were performed using GraphPad Prism 10 software (GraphPad Software, Boston, MA, USA).

## 4. Conclusions

Ultrasound-assisted extraction proved to be an effective method for isolating bioactive components from cloves and mandarin peels. The highest efficiency was observed for the innovative subsequent extraction, where cloves were extracted with mandarin peel extract as the solvent than during classic ethanolic extraction. The obtained extracts were successfully converted into dry powders by solvent evaporation followed by freeze-drying. Enrichment of rapeseed honey with 0.25% (*w*/*w*) of the dry clove and mandarin extracts significantly increased its antioxidant potential, indicating a high degree of functional enrichment. Research has confirmed that the adopted share of 0.25% is sufficient to impart new sensory characteristics of honey. However, the way of extract preparation was the key factor; the more active the extract, the greater the degree of enrichment. Honeys enriched with extracts demonstrated good stability of the analyzed parameters during storage for 6 months. Concluding, the addition of combined mandarin peel and clove dry extracts is a good way to improve the sensory characteristics of honey, and the resulting product may be considered as a functional food. However, further research on process optimization and biological activity assessment in terms of in vitro digestibility and bioaccessibility of introduced bioactive compounds are necessary before potential commercialization. The research indicates a new direction of enriching various types of food with phytochemical-rich dry extracts obtained according to the proposed novel method of extracting valuable raw materials.

## Figures and Tables

**Table 1:** Qualitative HPLC analysis of prepared extracts.

Retention Time [min]	Absorption Maxima [nm]	Tentative Identification	Presence in Extracts				
			M	C	M+C	p+C	b+M
2.20	218, 273	gallic acid *	−	+	+	+	+
2.33	295sh, 327	neochlorogenic acid *	+	−	−	+	+
3.02	291sh, 314	unknown phenolic acid	+	−	+	−	+
3.66	297sh, 327	caffeic acid derivative	+	−	+	−	+
4.11	297sh, 325	cryptochlorogenic acid *	+	−	+	−	+
4.52	280	unknown	−	+	−	−	−
4.93	293	unknown	−	+	+	+	+
5.12	295sh, 327	chlorogenic acid *	+	−	+	+	+
5.59	293	unknown	−	+	−	+	+
5.67	272, 334	apigenin glycoside	+	−	+	−	+
5.97	217, 274	syringic acid *	−	+	+	−	+
6.07	254, 272, 346	luteolin glycoside	+	−	+	−	+
6.23	368, 255	unknown	−	+	−	−	−
6.34	254, 272, 346	luteolin glycoside	+	−	+	−	−
6.36	368, 255	unknown	−	+	−	−	−
6.74	219, 273	gallic acid derivative	−	+	−	−	+
6.84	219, 273	gallic acid derivative	−	+	−	−	+
6.93	290, 326	unknown flavonoid	+	−	−	−	−
7.21	219, 273	gallic acid derivative	−	+	+	−	+
7.43	257, 269, 350	rutin *	+	−	+	+	−
7.44	219, 273	gallic acid derivative	−	+	−	−	−
7.52	257, 354	luteolin glycoside	+	−	−	−	−
7.53	219, 273	gallic acid derivative	−	+	−	−	−
7.79	254, 367	ellagic acid *	−	+	+	+	+
7.89	296sh, 324	caffeic acid derivative	+	−	+	+	+
7.91	257, 354	quercetin glycoside	−	+	+	−	+
8.04	272, 338	kaempferol glycoside	+	−	+	+	+
8.16	219, 273	gallic acid derivative	−	+	+	−	−
8.21	270, 330	apigenin glycoside	+	−	−	−	−
8.50	296sh, 327	caffeic acid derivative	+	−	+	−	−
8.53	266, 348	unknown flavonoid	−	+	−	−	+
8.67	285	hesperidin *	+	−	+	+	+
8.83	257, 274, 339	unknown flavonoid	+	−	+	−	+
8.96	255, 273, 335	unknown flavonoid	+	−	−	−	−
9.04	296sh, 328	caffeic acid derivative	+	−	−	−	−
9.14	296sh, 328	caffeic acid derivative	+	−	+	−	+
9.68	274, 331	unknown flavonoid	+	−	+	+	+
9.87	224, 276	unknown	−	+	+	+	+
10.13	275, 330	unknown	+	−	+	−	−
10.38	256, 271, 343	unknown	+	−	+	−	+
10.49	254, 267, 347	unknown flavonoid	−	+	−	+	+
10.61	276, 343	unknown flavonoid	+	−	+	−	+
10.77	320	unknown	+	−	+	−	+
11.11	254, 268sh, 347	luteolin *	+	−	+	+	+
13.31	268, 331	unknown flavonoid	+	−	+	−	−
13.51	231, 282	eugenol *	−	+	+	+	+
14.25	250, 271, 334	unknown	+	−	+	+	+
14.68	254, 271, 342	unknown flavonoid	+	−	+	+	+
15.25	271, 324	unknown	+	−	+	+	+
15.52	231, 282	eugenol derivative	−	+	+	+	+
15.85	236, 282, 329	unknown	−	+	+	+	+

* Identification confirmed by comparing the retention time with the standard, sh—band shoulder. M—mandarin peel extracted with 50% ethanol; C—clove buds extracted with 50% ethanol; M+C—mandarin peel and clove buds (1:1) extracted with 50% ethanol; b+M—clove buds extracted with M extract; p+C—mandarin peel extracted with C extract; (+) compound detected; (−) compound not detected.

**Table 2:** Physicochemical properties of pure (H) and enriched honeys after 1 month of storage.

Parameter	H	H_M+C_	H_b+M_	H_p+C_	H_M&C_
pH	4.89 ± 0.00	4.56 ± 0.00	4.54 ± 0.00	4.57 ± 0.02	4.57 ± 0.02
Titrable acidity [mval/kg]	20.30 ± 0.04	16.40 ± 0.06	15.00 ± 0.01 *	14.00 ± 0.02 *	15.60 ± 0.02
Electrical conductivity [µS/cm]	205.50 ± 0.50	227.25 ± 1.48	228.50 ± 2.69 *	226.00 ± 1.22	234.25 ± 4.26 *

(*) statistically significant differences (*p* < 0.05) compared to pure honey (H). H—control honey, H_M+C_—honey enriched with M+C dry extract, H_b+M_—honey enriched with b+M dry extract, H_p+C_—honey enriched with p+M dry extract, H_M&C_—honey enriched with combination M and C extracts in the ratio 1:1.

**Table 3:** Antioxidant properties (measured by DPPH and FRAP) and TPC in enriched honeys after 1-month storage.

Parameter	H	H_M+C_	H_b+M_	H_p+C_	H_M&C_
DPPH [μmol TE/100 g]	39.16 ± 13.35	145.17 ± 2.04 *	184.92 ± 6.57 *	136.50 ± 15.63 *	199.19 ± 9.09 *
Enrichment [%]	100	371	472	349	509
FRAP [μmol TE/100 g]	38.49 ± 6.51	295.02 ± 26.21 *	380.76 ± 6.98 *	254.73 ± 10.21 *	397.20 ± 4.60 *
Enrichment [%]	100	767	989	662	1032
TPC [mg GAE/100 g]	26.51 ± 5.66	92.35 ± 9.63 *	106.03 ± 3.60 *	87.52 ± 6.68 *	104.72 ± 5.61 *
Enrichment [%]	100	348	400	330	395

(*) statistically significant differences (*p* < 0.05) compared to pure honey (H). H—control honey, H_M+C_—honey enriched with M+C dry extract, H_b+M_—honey enriched with b+M dry extract, H_p+C_—honey enriched with p+M dry extract, H_M&C_—honey enriched with combination M and C extracts in the ratio 1:1.

**Table 4:** Qualitative HPLC analysis of 1-month stored honeys SPE extracts.

Retention Time [min]	Absorption Maxima [nm]	Tentative Identification	Presence in Extracts				
			H	H_M&C_	H_M+C_	H_p+C_	H_b+M_
3.59	297sh, 327	caffeic acid derivative	−	+	+	+	+
5.67	272, 334	apigenin glycoside	−	+	+	+	+
5.87	217, 274	syringic acid *	−	+	+	+	+
6.38	254, 272, 346	luteolin glycoside	−	−	+	+	+
7.33	257, 269, 350	rutin *	−	+	+	+	+
7.45	229, 311	p-coumaric acid *	+	+	+	+	+
7.60	254, 367	ellagic acid *	−	+	+	+	+
7.75	257, 354	quercetin glycoside	−	+	+	+	+
8.02	236, 294sh, 323	ferulic acid *	+	+	+	+	+
8.70	285	hesperidin *	−	+	+	+	+
9.42	232	benzoic acid *	+	+	+	+	+
9.81	296sh, 324	caffeic acid derivative	+	+	+	+	+
9.95	220, 276	methyl syringate *	+	+	+	+	+
11.16	254, 268sh, 347	luteolin *	−	+	+	+	+
11.76	268, 370	kaempferol *	+	+	+	+	+
11.98	292	pinobanksin *	+	+	+	+	+
12.48	218	unknown	+	+	+	+	+
13.51	231, 282	eugenol *	−	+	+	+	+
14.20	289	pinocembrin *	+	−	−	−	−
14.30	250, 271, 334	unknown	−	+	+	+	+
14.90	254, 271, 342	unknown flavonoid	−	+	+	+	+

* Identification confirmed by comparing the retention time with the standard, sh—band shoulder; compounds selected for quantitative analysis are marked in bold; (+) compound detected; (−) compound not detected.

## Data Availability

The original contributions presented in this study are included in the article. Further inquiries can be directed to the corresponding author.

## Supplementary Materials

The following supporting information can be downloaded at: https://www.mdpi.com/article/10.3390/molecules31091487/s1, Figure S1: HPLC-DAD chromatograms of analyzed extracts along with chromatogram for calibration standards; Figure S2: HPLC-DAD chromatograms of control honey and enriched honeys extracts prepared by SPE.; Table S1: HPLC method validation data.

## Abbreviations

The following abbreviations are used in this manuscript:

DPPH	2,2-Diphenyl-1-Picrylhydrazyl
FRAP	Ferric Reducing–Antioxidant Power
TPC	Total Phenolic Content
TE	Trolox Equivalent
GAE	Gallic Acid Equivalent
HPLC-DAD	High Performance Liquid Chromatography with Diode Array Detector
HPTLC	High Performance Thin Layer Chromatography
SPE	Solid-Phase Extraction
Rf	Retardation factor
ANOVA	Analysis of Variance
SD	Standard Deviation
