# Supplementary material for: Enrichment of Rapeseed Honey with Combined Polyphenol-Rich Dry Extracts from Mandarin Peels and Clove Buds
Source: Molecules. 2026 Apr 29;31(9):1487. doi: 10.3390/molecules31091487 (PMC13165162; doi:10.3390/molecules31091487)
Supplement: Supplementary file 1 [file molecules-31-01487-s001.zip › molecules-4247674-supplementary.pdf]

*Supplementary Material*

# The use of combined polyphenol dry extracts from mandarin fruit peels and clove buds to enrich rapeseed honey

**Dżugan M., Tomczyk M., Strzałka A., Miłek M.**

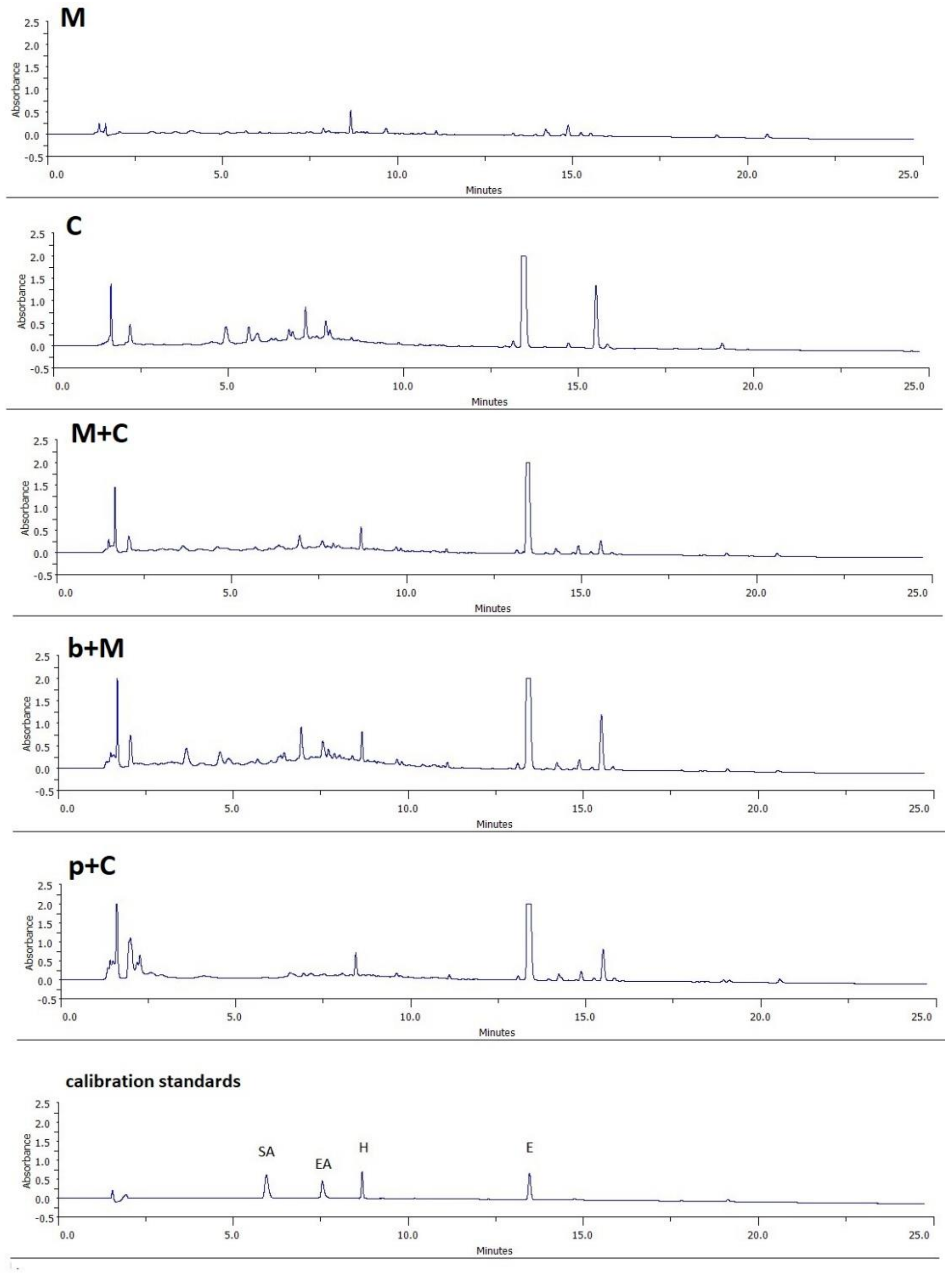


**Figure S1.** HPLC-DAD chromatograms of analyzed extracts along with chromatogram for calibration standards (SA – syringic acid, EA – ellagic acid, H – hesperidin, E – eugenol).
M - mandarin peel extracted with 50% ethanol; C - clove buds extracted with 50% ethanol; M+C - mandarin peel and clove buds (1:1) extracted with 50% ethanol; b+M - clove buds extracted with M extract; p+C - mandarin peel extracted with C extract.

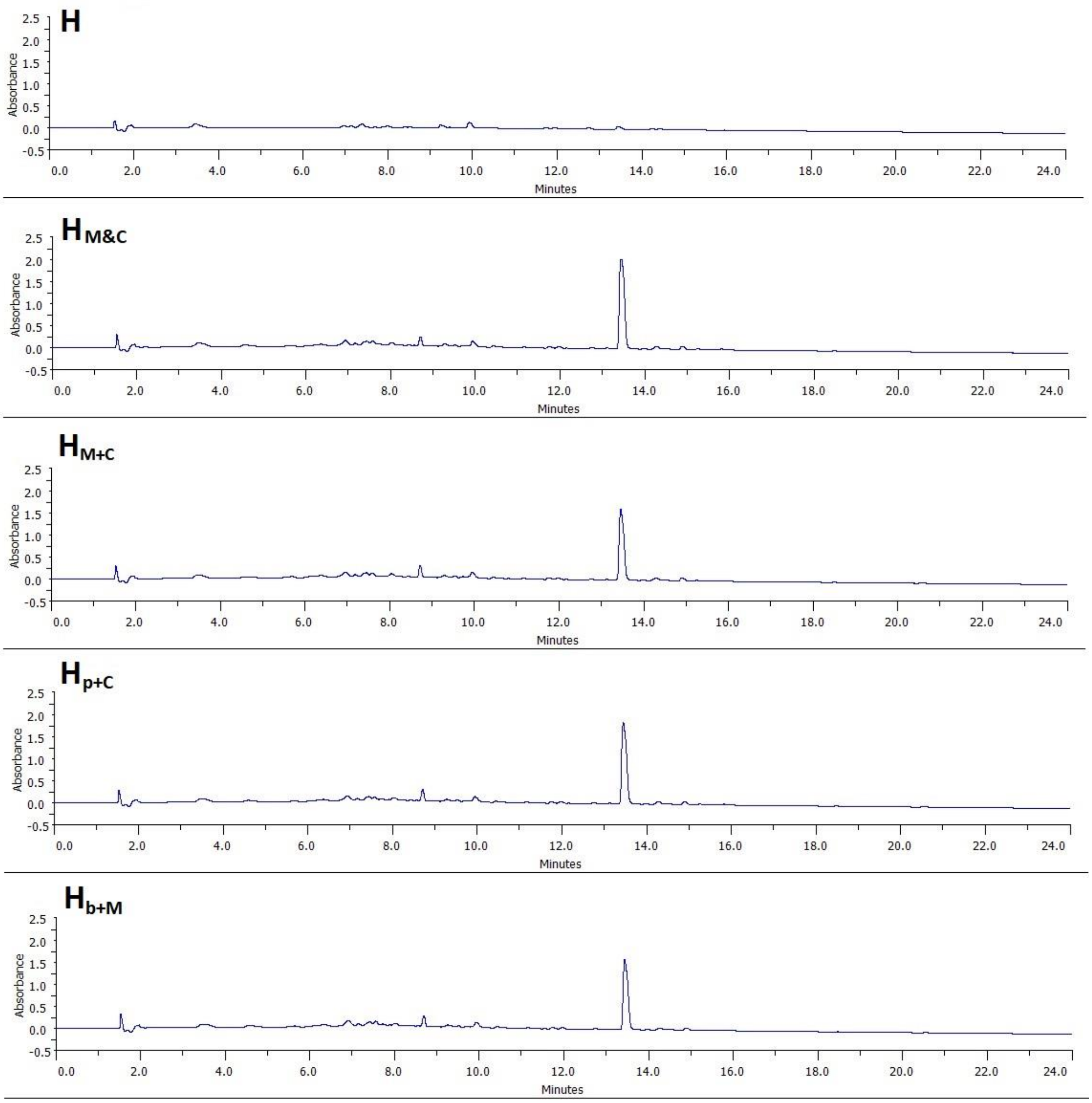


**Figure S2.** HPLC-DAD chromatograms of control honey and enriched honeys extracts prepared by SPE.

**Table S1.** HPLC method validation data.

| Compound | Retention time [min] | Standard curve equation | Linearity ($R^2$) | Calibration range [µg/ml] | LOD [µg/ml] | LOQ [µg/ml] |
|---|---|---|---|---|---|---|
| syringic acid | 5.96 | y = 27.788x + 282.29 | 0.9961 | 15.625 – 250.0 | 1.9 | 6.0 |
| ellagic acid | 7.79 | y = 18.895x – 218.16 | 0.9967 | 15.625 – 250.0 | 2.9 | 9.6 |
| hesperidin | 8.68 | y = 15.073 + 18.4 | 0.9985 | 15.625 – 250.0 | 2.0 | 6.6 |
| eugenol | 13.52 | y = 20.194x + 439.31 | 0.9955 | 15.625 – 250.0 | 0.77 | 2.5 |
